# A case of infective colitis due to *Yersinia enterocolitica* complicated by microliver abscesses mimicking multiple liver occult metastases: a case report

**DOI:** 10.1186/s12879-021-06177-6

**Published:** 2021-06-02

**Authors:** Rosario Luca Norrito, Chiara Pintus, Marco Cataldi, Alessandro Del Cuore, Mario Daidone, Valerio Vassallo, Maria Grazia Puleo, Tiziana Di Chiara, Salvatore Miceli, Giuseppina Maria Pizzo, Giuseppe Brancatelli, Antonino Tuttolomondo, Antonio Pinto

**Affiliations:** 1 U.O.C di Medicina Interna con Stroke Care, Dipartimento di Promozione della Salute, Materno-Infantile, di Medicina Interna e Specialistica di Eccellenza “G. D’Alessandro”, University of Palermo, Via del Vespro 129, 90127 Palermo, Italy; 2 Section of Radiology – BiND, University Hospital “Paolo Giaccone”, Via del Vespro 129, 90127 Palermo, Italy

**Keywords:** Microliver abscesses, Infective colitis, *Yersinia enterocolitica*, Case report

## Abstract

**Background:**

We report an unusual case of infective colitis by *Yersinia enterocolitica* complicated by microliver abscesses mimicking multiple liver metastases in a 79 yr old female without any risk factors for bacteriaemia by this pathogen.

**Case presentation:**

The patient was admitted to the Internal Medicine with Stroke Care ward of University Policlinico “P. Giaccone” in Palermo because of the appearance of diarrhoea. After the antimicrobial treatment for infective colitis, the clinicians observed a persistently increased white blood cells (WBC) count and multiple hepatic lesions; after having excluded any neoplastic disease and inflammatory bowel disease (IBD), blood cultures positive for *Y. enterocolitica* allowed to establish the final diagnosis was infective micro liver abscesses consequent to infective colitis due to *Y. enterocolitica*, which were successfully treated with cefixime and doxycycline.

**Conclusions:**

This case report should make clinicians reflect on how complex the differential diagnosis between microliver abscesses and metastasis could be and the possibility of bacteriaemia by *Y. enterocolitica* even without iron overload conditions.

## Background

The presence of multiple hepatic lesions can represent a challenge for clinicians. The differential diagnosis includes mainly neoplastic disease, especially metastases, and liver abscesses; consequently, it is necessary to examine both possible etiologies in parallel. The investigation of the possible presence of a neoplasm needs the use of imaging techniques, such as magnetic resonance imaging (MRI) and computed tomography (CT) scan; on the other hand, the infective hypothesis requires blood cultures and stool test to be confirmed. To establish the diagnosis, clinicians often need to obtain a biopsy, but it is not always feasible because of the smallness of the hepatic lesions or local expertise.

The typical symptomatology of liver abscesses reported in the literature is characterized by right upper abdominal pain, fever, jaundice and weight loss. Biliary infections represent the most common cause of bacterial liver abscesses [1], though other possible sources are IBD, bacteriaemia, hepatic traumas and suppurative appendicitis [2]. Enterobacterales, anaerobes [3] and Gram+ cocci are the most common pathogens involved in developing this pathology.

Other possible aetiologies are fungal infection (candida species) and parasitic one (*Entamoeba histolytica, Echinococcus granulosus)* [4].

We present the case of a woman affected by microliver abscesses mimicking metastases after infective colitis due to *Y. enterocolitica* apparently with no risk factors, such as haemochromatosis, thalassemias and chronic liver disease. The formation of abscesses from *Y. enterocolitica* is uncommon and usually consequent to the enteric disease, in particular terminal ileitis, mesenteric adenitis and enterocolitis.

## Case presentation

A 79 yr old Caucasian female affected by heart failure with preserved ejection fraction (HFpEF) and chronic kidney disease was admitted to the hospital because of diarrhoea associated with fever and griping abdominal pain that appeared just a few hours after a meal based on pasta with meat sauce. The patient took ciprofloxacin for three days as indicated by the general practitioner before being admitted to our ward. The patient was alert, oriented and afebrile; moderate pain in mesogastrium and hypogastrium after deep palpation was noted during the physical examination. Laboratory investigations (Table 1) revealed an increased WBC count with neutrophilia, raised reactive C protein (CRP) and faecal calprotectin.

**Table 1:** Results of laboratory investigations at admittance

*Heamoglobin*	*15.6 g/dL*
*WBC*	15850*10^*3*^*/μL*
*Neutrophils*	*82.2%*
*RCP*	*40.16 mg/L*
*Sodium*	*141 mEq/L*
*Potassium*	*4.82 mEq/L*
*Urea*	*48 mg/dL*
*Creatinine*	*1.21 mg/dL*
*Protein*	*64.9 g/L*
*Albumin*	*33 g/L*
*Total bilirubin*	*0.69 mg/dL*
*Alkaline phosphatase*	*85 U/L*
*Gamma-glutamyl transpeptidase*	*59 U/L*
*GOT/GPT*	*29/19 U/L*
*INR*	*1.1*
*Activated partial thromboplastin time*	*33 s*
*Serum iron*	*91 μ/L*
*Transferrin*	*312 mg/dL*
*Percentage of saturation of transferrin*	*33%*
*Ferritin*	*258 ng/mL*
*Faecal calprotectin*	*> 300 mg/Kg*
*CA 125*	*55.2 U/mL*
*CA 19–9*	*12.5 U/mL*
*β2-microglobulin*	*4.3 mg/L*
*CEA*	*2.03 μg/L*
*NSE*	*12 μg/L*
*α-fetoprotein*	*1.52 μg/L*

**Fig. 1:**
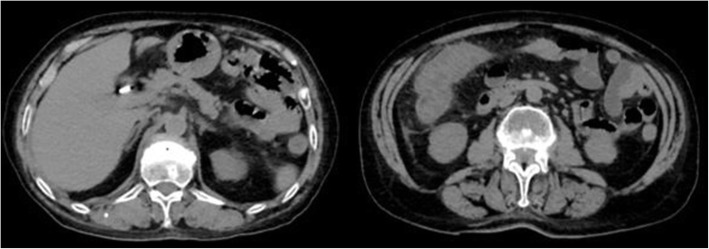
The abdomen TC scan performed at admittance revealed ubiquitous thickening of all the colic walls. ↑ RCP 40.16 mg/L - ↑ ESR 24 mm/h- ↑ WBC 15.85 × 10^3/μL - ↑ Faecal calprotectin > 300 mg/

The abdominal CT scan performed in the Emergency Department on the day before the admission in our ward showed thickening of all the colic walls with associated hyperdensity of the local adipose tissue and multiple enlarged mesenteric lymph nodes (Fig. 1)*.*

The patient was thought to be affected by infective colitis; consequently, clinicians required multiple blood cultures and stool tests before starting empiric antibiotic therapy with ciprofloxacin and metronidazole.

Initially, this therapeutic strategy seemed to be effective; in fact, the patient denied further diarrhoea episodes and the abdominal pain disappeared. Nevertheless, clinicians observed the appearance of a persistent low-grade fever (37.4–37-6 °C) and the blood tests showed an increase of markers of phlogosis, such as RCP (97.59 mg/L), and WBC count (20.69 * 10^3^/μL).

**Fig. 2:**
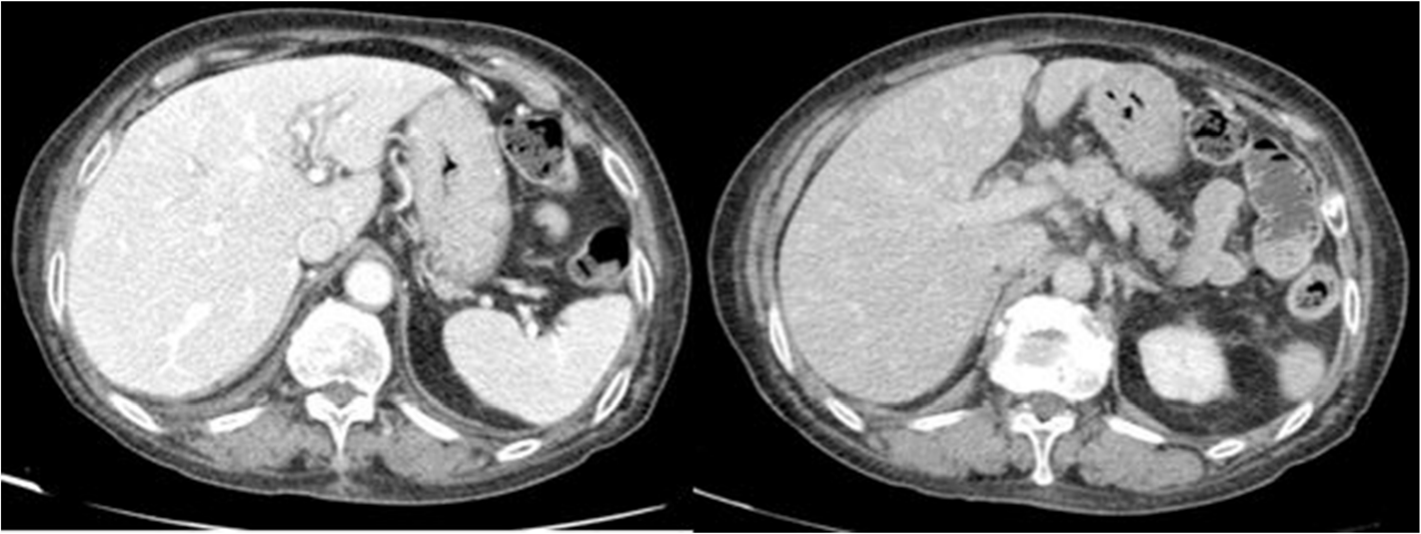
The second TC scan with medium contrast showed multiple small hypodense oval-shaded lesions provided with hyperdense borders during the arterial phase in the liver, hypodense during the portal and late phase. ↑ RCP 97.59 mg/L - ↑ WBC 20.69 × 10^3/μL

**Fig. 3:**
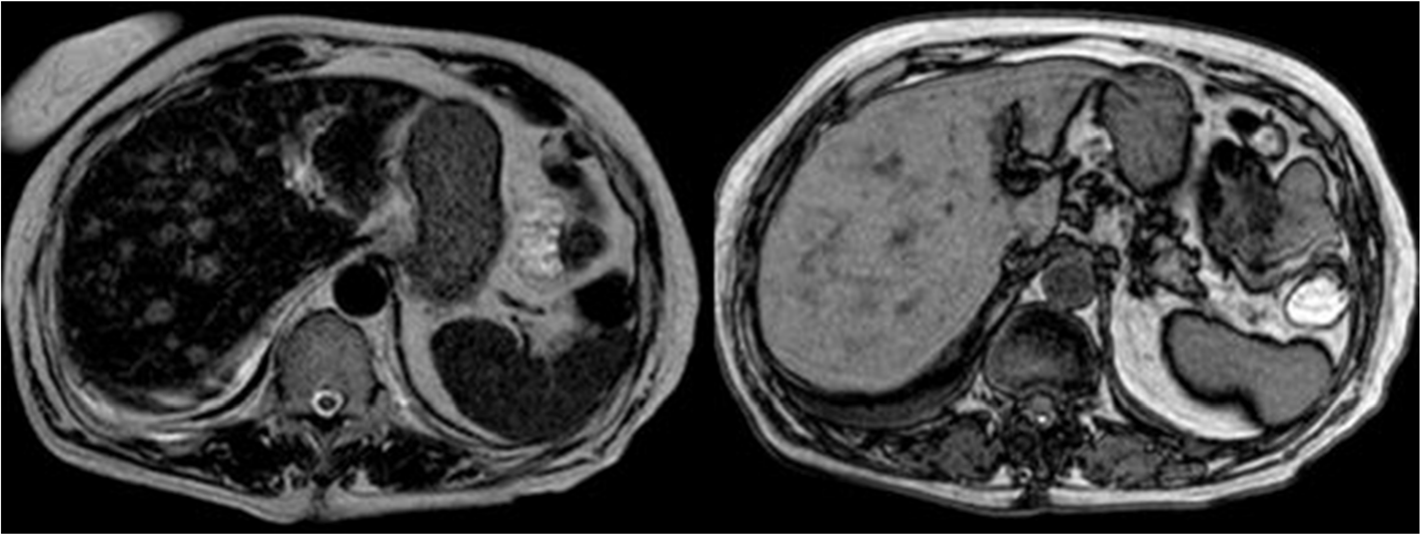
The abdominal MRI confirmed the presence of the lesions, which were hypointense in T1 weighted image and hyperintense in T2 one

Clinicians decided to repeat abdominal TC scan with contrast medium, which revealed multiple hypodense oval-shaded lesions provided with hyperdense borders during the arterial phase in the liver (Fig. 2). The abdominal MRI performed the next day confirmed the presence of these lesions that were hypointense in T1 weighted image and hyperintense in T2 one (Fig. 3), which were supposed to be the expression of metastatic disease by the radiologists.

The dosage of neoplastic markers was requested (CA 125, CA 19–9, ferritin, β2-microglobulin, CEA, NSE, α-fetoprotein), finding slightly increased ferritin levels, CA-125 and β2-microglobulin (Table 1)*.*

The patient underwent multiple colonoscopies that showed four benign adenomatous polyps and excluded the presence of lesions suggestive of neoplasms or IBD; the successive histological exam ruled out microscopic colitis.

Total body TC scan with contrast medium was performed searching for the possible primary neoplasm but resulted negative. Later, ^18^F-FDG - positron emission tomography (PET) did not show any lesion with altered metabolism, which is a possible finding suggestive of neoplastic disease. PET was preferred to MRI because of its ability to identify lesions with metabolic activity, such as metastases.

Because it was not possible to find any possible cancer with an imaging technique, the next step would have been the biopsy of one of the lesions to characterize them and identify the possible primitive site of the malignancy; unfortunately, the procedure was considered too dangerous for the patient because of the small size of the hepatic lesions and the consequent significant technique difficulty. Meanwhile, clinicians obtained stool tests and blood cultures (BACTEC 9120) results: while the firsts were negative, the second ones resulted positive for Gram-negative bacilli. In agar MacConkey lactose-negative colonies were observed, successive biochemical identification and antibiogram (MICROSCAN Walkaway - DADE Behering) identified *Y. enterocolitica* sensitive to doxycycline, gentamicin, ceftazidime and cefixime and resistant to amoxicillin/clavulanate and cotrimoxazole.

Consequently, specific antibiotic therapy with cefixime and doxycycline was started, resulting in the fever’s disappearance and the significant reduction of WBC count and RCP after a week *(*Table 2*).*

**Table 2:** Results of laboratory investigations at discharge

*Haemoglobin*	*11 g/L*
*WBC*	8750*10^*3*^*/μL*
*Neutrophils*	*59.3%*
*RCP*	*19.82 mg/L*
*Sodium*	*142 mEq/L*
*Potassium*	*3.68 mEq/L*
*Urea*	*68 mg/dL*
*Creatinine*	*0.98 mg/dL*
*GOT/GPT*	*31/25 U/L*
*Activated partial thromboplastin time*	*32 s*
*INR*	*1.19*

**Fig. 4:**
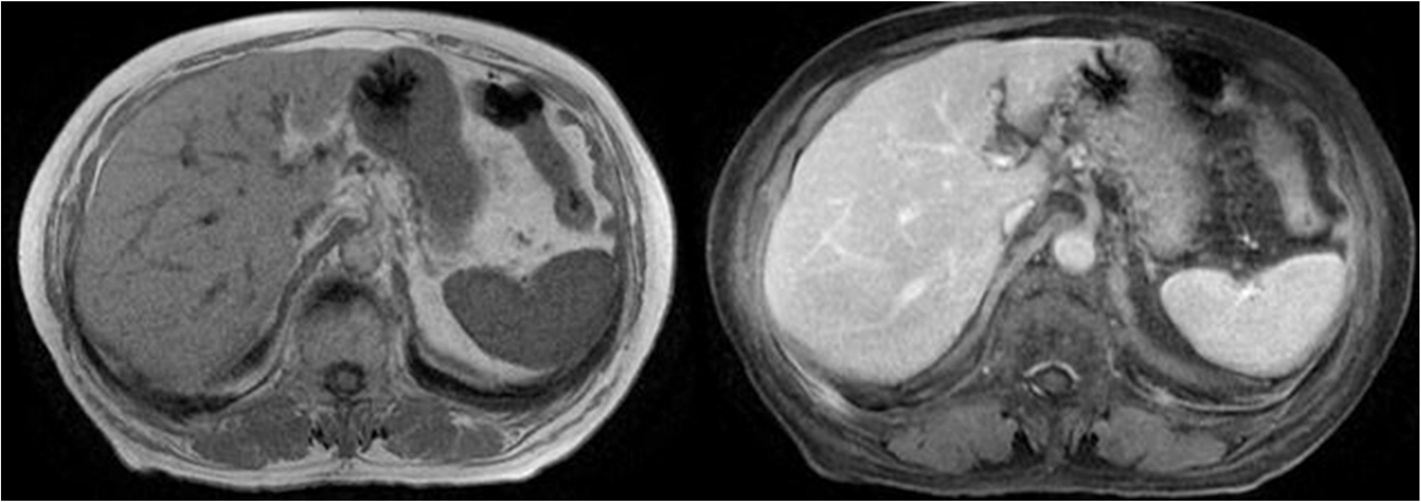
The MRI performed at three weeks showed the initial reduction of the lesions

**Fig. 5:**
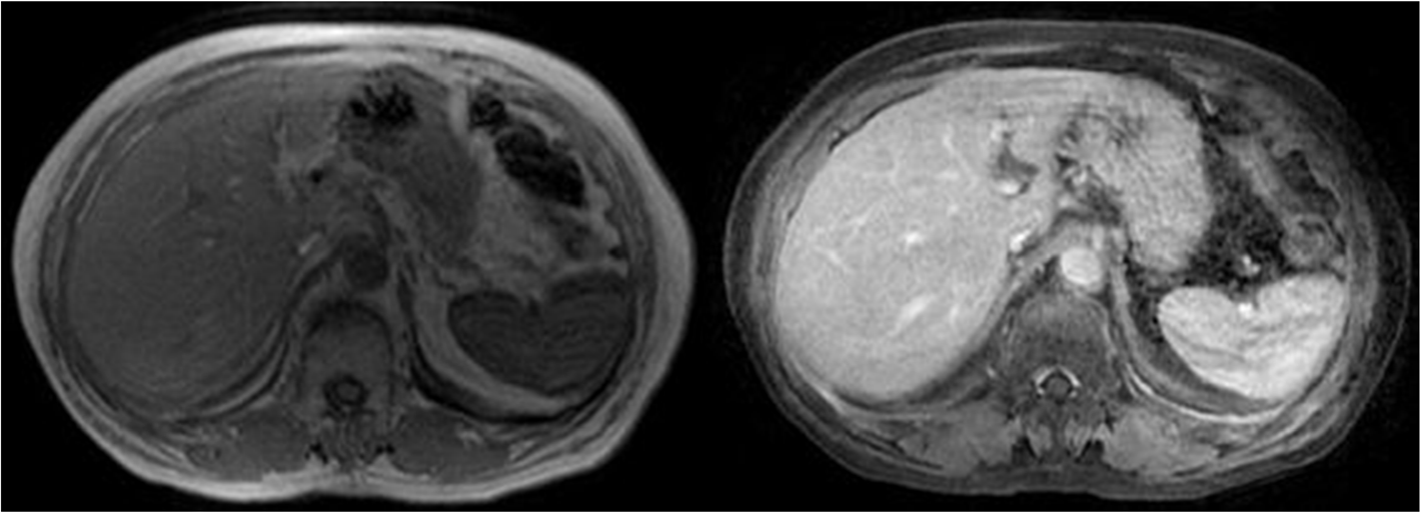
The MRI performed at 2 months showed the disappearance of the lesions

The patient continued this therapy for one month; abdominal MRI after three weeks (Fig. 4) and two months (Fig. 5) showed the initial reduction and the subsequent complete disappearance of the lesions.

Finally, blood culture and stool exam were both negative at two months.

Since microliver abscesses by *Y. enterocolitica* are strictly associated with haemochromatosis, clinicians excluded this pathology by liver MRI and the percentage of transferrin saturation.

## Discussion and conclusion

Multiple liver lesions could often be the expression of either hepatic metastases or abscesses or cysts; the differential diagnosis is not always straightforward.

Imaging techniques sometimes do not manage to be conclusive for the diagnosis; while cysts have a typical radiological pattern difficult to be confused, on the other hand, the distinction between metastases and microliver abscesses can be intricate, especially when patients have no previous neoplastic disease. Even the laboratory investigations could not be diriment: significantly increased white blood count and markers of inflammation are suggestive of an infective aetiology, but it is possible to observe borderline levels of these markers, which is the most common situation, both in hepatic metastases and microliver abscesses.

*Y. enterocolitica* is a gram-negative coccobacillus and a member of *Enterobacterales.* This bacterium grows in non-selective agar at a temperature between 25 °C and 37 °C; it ferments sucrose and glucose but not lactose and is oxidase negative [5].

The *yersinioses* are zoonotic infections of wild and domestic animals; humans are usually incidental hosts [6]*.*

Infection due to *Y. enterocolitica* is a cause of enterocolitis or mesenteric lymphadenitis, a pathology with a clinical presentation similar to appendicitis. In up to 30% of subjects, post-infectious sequelae, typically glomerulonephritis, erythema nodosum or Reiter’s syndrome, are possible [7].

Most subtypes of *Y. enterocolitica* lack effective methods for iron uptake [8]; just a few strains have some genes for an iron-binding protein called *yersiniabactin* [9]. This fact explains why septicemia by *Y. enterocolitica* is rare in patients without pathologies characterized by an iron-overload such as haemochromatosis, thalassemias and chronic liver disease.

The diagnosis of infection by *Y. enterocolitca* is microbiological, isolating bacteria from biological samples, such as blood and faeces.

The treatment for liver abscesses due to *Y. enterocolitica* is typically medical, and it is based on antibiotic therapy with ciprofloxacin, ceftriaxone, cotrimoxazole or gentamicin for at least three weeks; drainage is possible only in solitary abscesses.

This clinical case began with common infective colitis and evolved into a complex one because of multiple reasons that delayed the final diagnosis. First of all, the hepatic lesions’ radiological characteristics were suggestive for metastatic disease; consequently, clinicians spent time and resources searching for primitive cancer. A biopsy would have quickened the diagnostic process, but unfortunately, the lesions’ small size made the procedure too tricky and dangerous. Furthermore, the development of liver abscesses as a complication of infective colitis by *Y. enterocolitica* is rare. As mentioned above, bacteriaemia is improbable without systemic pathologies characterized by a condition of iron-overload; since the patient did not have any risk factor, the presence of microliver abscesses due to *Y. enterocolitica* was unexpected, making this case rather peculiar.

A possible explanation of the initial absence of fever and the negativity of the stool tests could be represented by the ciprofloxacin taken by the patients before hospital admittance; this therapy could have been partially effective, but it did not avoid bacteriaemia and the subsequent formation of microliver abscesses which caused the appearance of fever and the increase of the markers of inflammation.

The diagnosis was the result of effective multidisciplinary teamwork between internists, radiologists and microbiologists.

In this case, we observed how challenging the differential diagnosis between hepatic metastases and microliver abscesses could be; furthermore, bacteriaemia by *Y. enterocolitica* cannot be excluded in patients with suggestive symptomatology even when there are no risk factors.

## Data Availability

Data sharing is not applicable to this article as no datasets were generated or analysed during the current study.

## Abbreviations

WBC: White blood cells
IBD: Inflammatory bowel disease
MRI: Resonance imaging
CT: Computed tomography
HFpEF: Heart failure with preserved ejection fraction
CRP: Reactive C protein
^18^F-FDG: Fluorodeoxyglucose F 18
PET: Positron emission tomography
